# 3D Imaging of Mitral Valve Perforation as a Complication of Austrian Syndrome

**DOI:** 10.1111/echo.70285

**Published:** 2025-09-04

**Authors:** Nitya Panyala, Kyle Rusin, Nathan D. Wheeler

**Affiliations:** ^1^ Indiana University School of Medicine Ball Memorial Muncie Indiana USA; ^2^ Indiana University School of Medicine Indianapolis Indiana USA; ^3^ Department of Cardiology Indiana University School of Medicine IU Health Indianapolis Indiana USA

**Keywords:** Anterior leaflet perforation, Austrian syndrome, Mitral valve vegetation, Severe mitral regurgitation

## 1 Description

The following are echocardiographic findings in a 68‐year‐old male with Austrian syndrome.

The patient presented with sinus drainage, headache, fever, mild productive cough, and conjunctivitis, all of which worsened over 2 weeks. Physical exam revealed a holosystolic murmur in the apex radiating to the axilla, and bilateral rales on auscultation. The work‐up revealed multidrug‐resistant Streptococcus pneumoniae bacteremia, meningitis, and right eye endophthalmitis.

**FIGURE 1:**
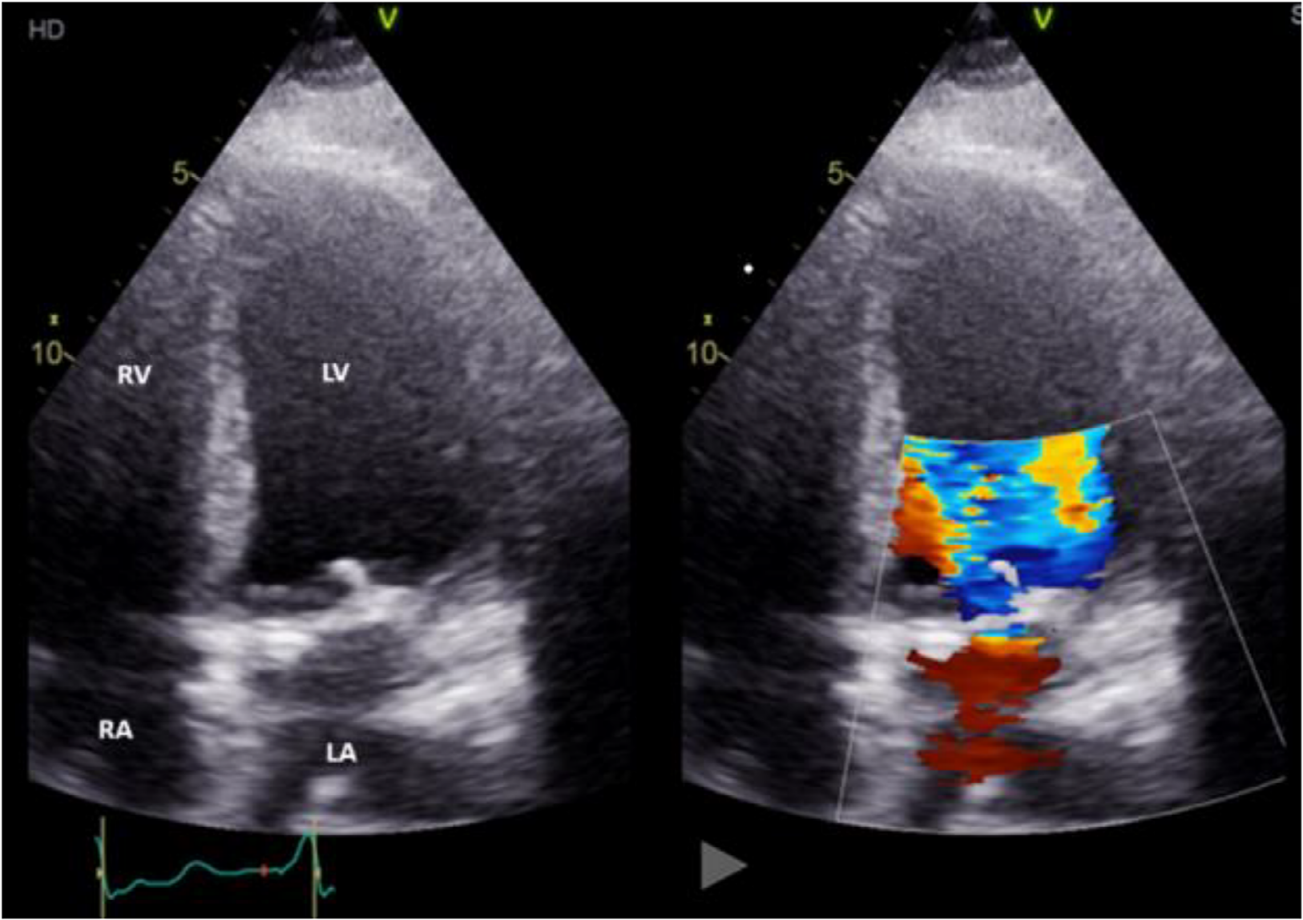
Transthoracic echocardiogram showing severe mitral regurgitation in apical four‐chamber view. The right panel shows color Doppler imaging with turbulent regurgitant flow (blue and orange jets) across the mitral valve into the left atrium. LA, left atrium; LV, left ventricle; RA, right atrium; RV, right ventricle.

Initially, a transthoracic echocardiogram (TTE) was ordered to determine if there was any valvular involvement. TTE findings confirmed mitral valve vegetation and quantified the mitral regurgitation (MR) as severe (Figure 1). Measurements included a peak MR gradient of 71.4 mmHg, an MR volume of 83.9 mL, and an MR effective regurgitant orifice area (EROA) of 0.8 cm^2^. The MR volume and EROA were measured using the proximal isovelocity surface area (PISA) method [1].

**FIGURE 2:**
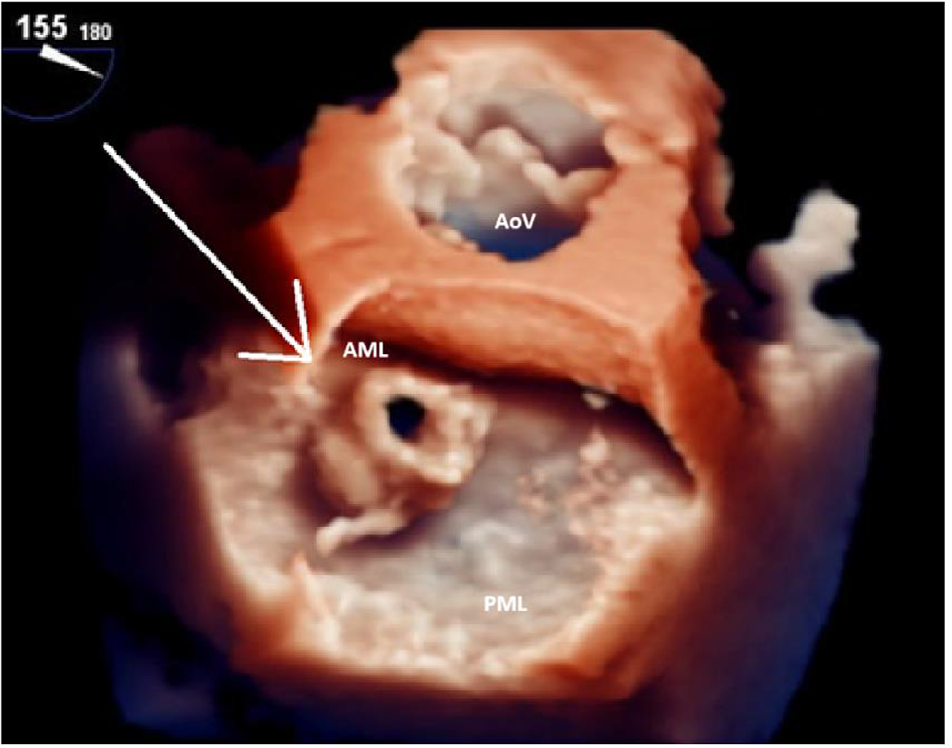
Three‐dimensional transesophageal echocardiogram demonstrating a large, irregular vegetation (white arrow) attached to the mitral valve with associated perforation of the anterior mitral leaflet. AML, anterior mitral leaflet; AoV, aortic valve (opened); PML, posterior mitral leaflet (closed).

**FIGURE 3:**
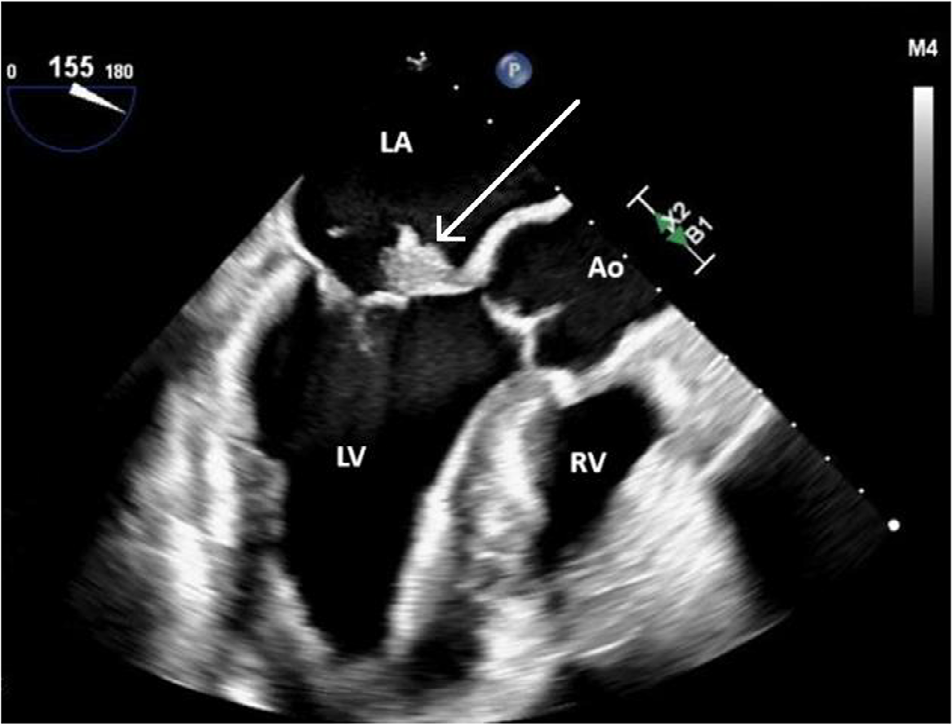
Transesophageal echocardiogram demonstrating a large, echodense vegetation (white arrow) attached to the anterior leaflet of the mitral valve. Ao, proximal ascending aorta; LA, left atrium; LV, left ventricle; RV, right ventricle.

**FIGURE 4:**
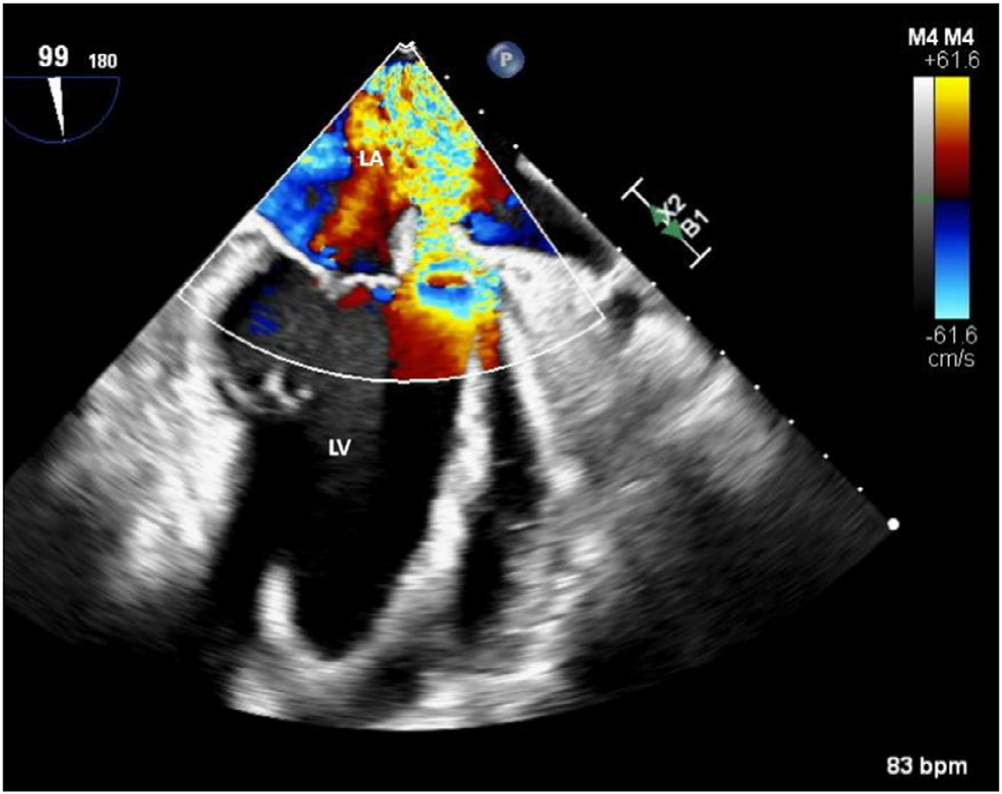
Transesophageal echocardiogram with color Doppler demonstrating severe mitral regurgitation secondary to valvular destruction from pneumococcal endocarditis. LA, left atrium; LV, left ventricle.

Transesophageal echocardiography (TEE) was performed to obtain a detailed view of the vegetation and to detect any other potential structural abnormalities. A large vegetation was seen on the mitral valve with associated anterior leaflet perforation (Figures 2, 3), resulting in severe regurgitation (Figure 4). These findings represented advanced valvular destruction, a hallmark of aggressive pneumococcal endocarditis.

The constellation of pneumococcal endocarditis, meningitis, and pneumonia established the diagnosis of Austrian syndrome, a rare but classical triad associated with invasive pneumococcal disease [2].

The confirmation of a massive mitral valve vegetation with anterior leaflet perforation established the anatomical basis for severe regurgitation, thus requiring surgical intervention. The patient responded favorably to antibiotic therapy with eventual clearance of bacteremia. Following clinical stabilization, cardiothoracic surgery was consulted, and the patient subsequently underwent successful mitral valve replacement.

This case is notable for its devastating echocardiographic findings related to Austrian syndrome, which revealed a massive vegetation on the mitral valve. The vegetation caused a perforation extending through both the vegetation and the anterior leaflet. This case illustrates the highly destructive nature of pneumococcal endocarditis, which can rapidly erode cardiac tissue and necessitate emergency surgical intervention.

## Data Availability

Data sharing is not applicable to this article as no datasets were generated or analyzed during the current study.
